# The Necrobiology of Mesenchymal Stromal Cells Affects Therapeutic Efficacy

**DOI:** 10.3389/fimmu.2019.01228

**Published:** 2019-06-04

**Authors:** Daniel J. Weiss, Karen English, Anna Krasnodembskaya, Johana M. Isaza-Correa, Ian J. Hawthorne, Bernard P. Mahon

**Affiliations:** ^1^Department of Medicine, University of Vermont College of Medicine, Burlington, VT, United States; ^2^Cellular Immunology Laboratory, Biology Department, Human Health Research Institute, Maynooth University, Maynooth, Ireland; ^3^School of Medicine, Dentistry and Biomedical Sciences, Wellcome-Wolfson Institute for Experimental Medicine, Queen's University of Belfast, Belfast, United Kingdom; ^4^Immunology & Cell Biology Laboratory, Biology Department, Human Health Research Institute, Maynooth University, Maynooth, Ireland

**Keywords:** mesenchymal stromal cell, cell therapy, apoptosis, autophagy, mitochondria, extracellular vesicles, efficacy

## Abstract

Rapid progress is occurring in understanding the mechanisms underlying mesenchymal stromal cell (MSC)-based cell therapies (MSCT). However, the results of clinical trials, while demonstrating safety, have been varied in regard to efficacy. Recent data from different groups have shown profound and significant influences of the host inflammatory environment on MSCs delivered systemically or through organ-specific routes, for example intratracheal, with subsequent actions on potential MSC efficacies. Intriguingly in some models, it appears that dead or dying cells or subcellular particles derived from them, may contribute to therapeutic efficacy, at least in some circumstances. Thus, the broad cellular changes that accompany MSC death, autophagy, pre-apoptotic function, or indeed the host response to these processes may be essential to therapeutic efficacy. In this review, we summarize the existing literature concerning the necrobiology of MSCs and the available evidence that MSCs undergo autophagy, apoptosis, transfer mitochondria, or release subcellular particles with effector function in pathologic or inflammatory *in vivo* environments. Advances in understanding the role of immune effector cells in cell therapy, especially macrophages, suggest that the reprogramming of immunity associated with MSCT has a weighty influence on therapeutic efficacy. If correct, these data suggest novel approaches to enhancing the beneficial actions of MSCs that will vary with the inflammatory nature of different disease targets and may influence the choice between autologous or allogeneic or even xenogeneic cells as therapeutics.

## Introduction

The efficacy of MSC administration in preclinical inflammatory models is well-documented regardless of the source of MSCs (bone marrow, adipose, placenta, other). The basic biology of MSCs, their mode of action and therapeutic efficacy in clinical studies have been reviewed extensively elsewhere (1–3). However, translation of preclinical efficacy to the clinical setting is proving difficult. A possible reason for this is a lack of understanding of the fate of MSCs when they encounter highly inflammatory microenvironments. Within this inflammatory milieu, MSCs are exposed to insults such as hypoxia and pro-inflammatory cytokines (4). What happens to MSCs during the transient period in which they are at the target site is largely unknown (5). The longstanding working hypothesis has been that viable functional MSCs are critical for efficacy. However, a number of recent studies have suggested that MSC survival in the disease milieu may not be as important as once thought. These studies elegantly demonstrate that apoptotic or dead MSCs can facilitate protection mediated by MSC administration in inflammatory microenvironments *in vivo* (6–8). However, these studies have opened up a number of questions about the processes involved in the transition from live to dead MSCs. Under what circumstances can dead MSCs substitute for viable cells? What are the limits to use? Can the pre-apoptotic cargo of extracellular vesicles (EVs) produced by MSCs or mitochondria transferred from MSCs to other cells substitute for the MSCs themselves? Is there a role for autophagy or for efferocytosis in MSCT efficacy? Does autophagy influence the soluble factors secreted by MSCs before they die? If we can better understand the fate of MSCs within the diseased microenvironment, perhaps this knowledge would lend itself to development of more optimal MSC-based cell therapies (be that live, autophagic or dead/apoptotic MSCs) and reduce the disparity between pre-clinical models and the clinical setting.

**Figure 1:**
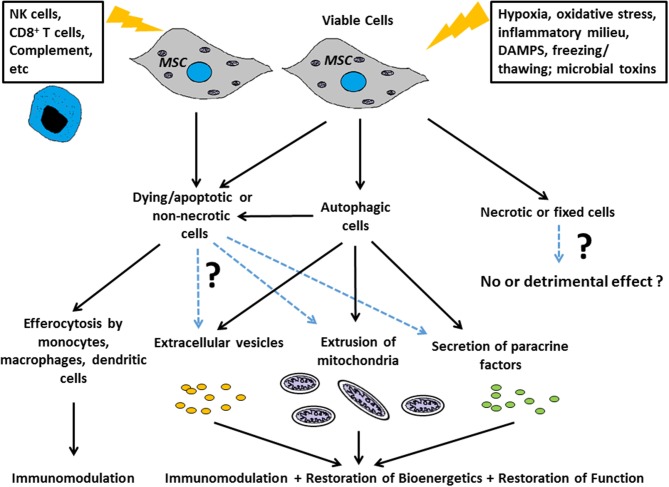
Scheme for how the necrobiology of MSCs influences therapeutic efficacy Putative mechanisms include: as live cells through paracrine mechanisms, and through the cellular processes associated with morphological, biochemical, and molecular changes which predispose, precede, and accompany cell death. These necrobiotic processes include the response to dying and non-necrotic MSCs, the alteration of MSC biology by autophagy, and the delivery of MSC derived mitochondria or EVs to target cells and tissues.

The term “necrobiology” has been used to describe the cellular processes associated with morphological, biochemical, and molecular changes which predispose, precede, and accompany cell death, as well as the consequences and tissue response to cell death (9). The observation that MSC viability and efficacy are not necessarily correlated (6, 7, 10) suggests that the necrobiology of MSCT will be a fruitful and essential area for future study. In this review we focus on key biological processes likely to affect therapeutic efficacy (Figure 1), summarize what is known about the questions above, and for the first time attempt to frame these disparate aspects of research within the concept of necrobiology or the biology of the dying therapeutic cell.

## Apoptotic MSCs and Clinical Efficacy

There is relatively little data available in pre-clinical disease models in which apoptotic or dead MSCs were investigated, either as part of a direct investigation of dead/apoptotic cell actions or as part of a control group for live MSC administrations. Using pre-clinical models of respiratory diseases/critical illnesses in mice as representative examples (Table 1), intratracheal administration of apoptotic MSCs in models of acute lung injury or systemic administration of either fixed or heat-killed MSCs in mouse models of asthma and sepsis, respectively, did not mimic the effects of live MSC administration (11–14). Likewise the administration of other cells such as fixed fibroblasts were not beneficial, suggesting a role for MSCs that cannot be replaced by other dead cell types (11, 13). Notably, most of these studies are relatively old and did not exhaustively explore the effects of dead or apoptotic cells on immune or inflammatory cells. Whether this is a phenomenon unique to MSCs is unknown at present as there are few examples of administering other types of cells to the lung that might influence inflammatory or immune pathways. However, there are well documented anti-inflammatory bystander effects when other apoptotic cells are engulfed by macrophages and these have been recently reviewed (15). The extent to which this phenomenon is specific to lung diseases is relatively unexplored and a ripe area for further research.

**Table 1:** Pre-clinical lung injury studies utilizing dead or apoptotic MSCs.

**Injury Model**	**Experimental model, route, and timing of treatment**	**MSC Source**	**Outcome compared to injury effects**	**Potential mechanisms of MSC actions**	**Cell controls?**	**References**
Acute Lung Injury	Mouse IN LPS IT MSC 4 h after LPS	Syngeneic Mouse BM Plastic Adherent	Improved survival Improved histologic inflammation and edema Decreased BALF TNF-α, MIP-2 Increased BALF and serum IL-10	None specified (soluble mediators)	Apoptotic MSC, 3T3 fibroblasts Did not mimic effects on survival or inflammation	(11)
Acute Lung Injury	Mouse IT LPS IT MSC 4 h after LPS (P 5–6); 10^6^ cells/mouse	Xenogeneic Primary human umbilical cord MSC CD29+, 44+, 73+. CD34-, 45-, HLAII- osteo/adipo differentiation	Decreased mortality, histological injury (3d), BAL TNFa, MIP-2, IFNγ (3d), Th1 CD4 T cells Increased BAL IL-10 (3d), CD4/CD25/Foxp3+ Treg	Non-specified soluble mediators	Apoptotic MSCs (mitomycin C treated) Did not mimic MSC results	(12)
Asthma	Mouse ovalbumin-induced acute allergic airways inflammation Ovalbumin sensitization days 0, 7, 14 MSC IV days 7/14 (P 4–9), 5 × 10^6^ cells/infusion Challenge days 25–27; Harvest d28	Allogeneic Mouse (FVB) BM Sca1+, CD44+, 106+. CD11b-, 11c-, 34-, 35-, 117- Osteo/adipo/ Chondro differentiation	Decreased histological injury, BAL total cells (especially Eosinophils & Macrophages), BAL IL-4, IL-13, splenocyte IL-4 recall Increased BAL IL-10; splenocyte IL-13, IL-10 recall	None (paracrine)	PFA-fixed MSC Did not mimic most MSC results No effect or exacerbated histology; no effect on BAL except BAL IL-13; Increased splenocyte IL-4 recall. Decreased splenocyte IL-10, IL-13 recall.	(13)
Sepsis	Mouse cecal ligation and puncture IV MSC 1 h prior, concomittant, or 24 h after surgery	Syngeneic & allogeneic Mouse BM Plastic adherent CD11b, 45 depleted	Improved survival and organ function Decreased circulating TNFα, IL-6 Increased circulating IL-10	LPS and TNFα-stimulated MSC stimulated macrophages produced IL-10 through cell-cell contact and iNOS-dependent release of PGE2	Whole bone marrow, heat-killed MSC, skin fibroblasts No effects on survival Other endpoints not assessed	(14)

In contrast, more recent studies in pre-clinical models of acute lung injury have suggested that the inflammatory environment in the lung can affect survival and subsequent efficacy of intratracheally-administered MSCs in part through activation of TRL4 signaling pathways (16). MSCs have variable effects in different mouse models of lung injuries with efficacy potentially related to the proteome profile of the BAL fluid in each respective injury (17). Another recent study demonstrated that apoptotic MSCs reduced some inflammatory endpoints in a mouse model of Th2-mediated allergic airway inflammation (7). These effects are not confined to lung disease models, a series of related studies in a rat model of cecal ligation and puncture-induced sepsis demonstrated that administration of rat adipose-derived MSCs, rendered apoptotic by 96 h culture in serum-free media, were more effective than healthy MSCs in improving survival and decreasing lung, kidney and cardiac injuries (18–21) administration of the apoptotic MSCs decreased a number of circulating and organ-specific pro-inflammatory, pro-apoptotic, and oxidative stress markers while increasing anti-apoptotic and anti-oxidant responses. The suggested mechanism(s) were that the apoptotic MSCs were more effective at dampening immune responses to the original injury, however, no specific pathways were delineated. These results suggest a more complex interaction of MSC apoptosis on efficacy in different inflammatory environments such that the inflammatory environment itself directs MSC apoptosis. Unfortunately, other more recent studies of MSC effects in a wide range of pre-clinical lung injury models have generally not included dead or apoptotic cells and thus there is opportunity for more extensive investigation (22–25).

Surprisingly, little is known about how MSCs are killed in different settings. *In vitro* studies have demonstrated the conditions for NK cell killing of MSCs (26) and this is likely to be an important mechanism for induction of MSC death *in vivo*, although few studies have examined this in detail. Similarly a role for Complement mediated killing has been proposed (27, 28). Recently, a requirement for cytotoxic CD8^+^ T cell mediated killing (via apoptotic death) of MSCs has been shown in GvHD (7). However, the mechanisms of MSC killing (e.g., immune-mediated or as a result of exposure to microbial toxins) are likely to influence the type of death induced and the biological consequence. This might be an especially important consideration in designing cell therapeutics for lung diseases or patient subsets where there is a pathogenic microbial burden (e.g., Cystic Fibrosis).

There are even less available data on the effect of apoptotic MSCs in clinical investigations. In a notable recent example, safety but no efficacy was observed in a multi-center double-blinded randomized trial of systemic bone marrow-derived MSCs in patients with ARDS (29). In *post-hoc* analyses, the unanticipated finding was that up to 85% of the MSCs were non-viable at the time of administration. This suggests that dead MSCs may not have clinical efficacy in ARDS although there are a number of other factors to consider including timing, dose, and route of MSC administration (30). In contrast, a preliminary report of a parallel trial of bone marrow-derived MSC-like cells in ARDS patients demonstrated efficacy in major endpoints of survival, ventilator-free days and ICU stay (31). Notably, the cells utilized were fully viable at the time of administration. Therefore, viable/ live MSCs are not interchangeable therapeutically with apoptotic/dead MSC, but each have potential efficacy in different contexts and presumably by different mechanisms. In combination with the growing experience of dead/apoptotic MSCs in pre-clinical models, these clinical observations raise important and hypothesis-generating mechanistic ideas for further study.

## MSC Autophagy and Clinical Efficacy

It is now known that non-necrotic cell death can be induced by diverse mechanisms and many of these are linked to the cellular processes that eliminate damaged proteins and organelles, termed autophagy (32, 33). Autophagy is a tightly regulated, complex cascade that controls the efficient delivery and fusion of damaged organelles to the autophagosome (34). Whilst this process supports cell survival and regular cell functioning, it is also associated with at least three forms of cell death- apoptosis, necroptosis, and autosis. Necroptosis is an inflammatory, caspase-independent form of cell death (33) whereas autosis is mediated by the Na^+^ K^+^- ATPase pump and is autophagy-gene dependent (35, 36). More broadly, autophagy is activated by microenvironmental and intracellular signals linked to ER stress, hypoxia and immune cell activation (37–39). These signals, related to tissue damage, include damage associated molecular patterns (DAMPs) and MSCs have been shown to sense DAMPs released from dying/stressed cells (40) leading to enhanced pro-reparative and anti-inflammatory effects (40). Thus, the reparative effects of MSCs may be primed or altered by exposure to DAMPs or other stress signals that alter their interactions with other cells. During cell therapy, MSCs become exposed to such signals and autophagy is a common cellular response to such stress. Autophagy influences MSCs' therapeutic effects in at least two contrasting ways- to promote survival of the MSCs, or to induce MSC death through apoptosis, necroptosis, or autosis. The fate of the MSC is thus likely to be dependent on quantitative differences in exposure time to inflammation. Understanding the role of autophagy in MSCs at sites of inflammation could therefore inform therapeutic protocol design in the future.

The role played by autophagy as a survival mechanism to relieve stress and prevent apoptosis has been extensively studied (41). Under starvation conditions (serum deprivation, hypoxia, oxygen/glucose deprivation) or in the presence of reactive oxygen species (ROS), autophagy has been shown to promote MSC survival *in vitro* (42–44). Importantly, while sufficient levels of ROS are required to activate autophagy, excessive ROS may lead to cell death (45). This has been demonstrated where preconditioning of MSCs to serum deprivation and hypoxic conditions have prolonged survival in ischemic microenvironments through the activation of autophagic processes (46). Moreover, mitophagy in MSCs facilitates interaction with macrophages in conditions of oxidative stress whilst also preventing apoptosis (47). A number of extrinsic factors that modulate autophagy in MSCs have been identified, for example Stromal Cell Derived Factor-1β can promote MSC survival through enhanced autophagy (48). Expression of hypoxia-inducible factor 1α also protects against Oxygen-Glucose deprivation via induction of autophagy and the PI3K/AKT/mTOR signaling pathway (43), while over-expression of CPT1C in human MSCs enhances survival via an increase in autophagic flux (49). In aging mice, knockdown of insulin-like growth factor-1 enhances survival of MSCs through autophagy and prolongs MSC survival *in vivo* (50). Taken together these studies clearly show that at least in some circumstances autophagy promotes MSC survival under stress.

In addition to factors influencing the autophagic pathway in MSCs, autophagy may also lead to the production of soluble factors important for MSC's therapeutic efficacy. Vascular endothelial growth factor (VEGF) plays a key role in MSCs promotion of wound healing (51, 52), a recent study has identified that increased VEGF secretion from autophagic MSCs promoted vascularization in cutaneous wounds and improved healing (52). The induction of autophagy in MSCs may also alter their immunomodulatory function. Autophagic human bone marrow-derived MSCs can regulate CD4^+^ T helper cell proliferation via TGF-β1 signaling (53). Activation of autophagy by rapamycin in a co-culture system enhanced MSC's ability to suppress CD4^+^ T helper cell proliferation, whilst 3-methyladenine (3-MA), an autophagic inhibitor, reduced it. These data indicate a role for autophagy in MSCs' immunomodulatory functions of the adaptive immune response, and therefore suggest that the autophagic status of the MSCs will influence therapeutic efficacy under inflammatory conditions. However, the precise limits of this effect are unknown and there are clearly redundant and parallel mechanisms operating. For example, Chinnadurai et al. showed that while interferon gamma (IFN-γ) stimulation of MSCs upregulated the expression of autophagy genes, inhibition of autophagy via 3-MA did not affect MSCs' immunomodulatory potential (54). Furthermore, in some studies, autophagy was shown to have adverse effects on MSCs' immunomodulatory capacity. When rodent MSCs were stimulated with tumor necrosis factor (TNF) and IFN-γ, autophagy reduced MSCs' immunomodulatory effects whereas inhibition through the knockdown of Bcn1 was beneficial (55). It is difficult to compare studies that inhibit autophagy when diverse inducers and inhibitors have been used, or when different inhibitor concentrations and time points have been studied. Nevertheless, these differences are important, autophagy and indeed apoptosis are time dependent processes, and it is reasonable to assume that the activity and function of the MSCs transitioning through these processes will vary with each disease and over time. The implication for developing therapies is that future preclinical approaches will have to account more comprehensively for temporal and dose effects to be informative, but such information could well-shorten therapeutic development times if it leads to improved understanding of delivery route and dosage.

Autophagy can alter biological function following starvation or inflammation (56), and in contrast to the above, can promote autophagic cell death or autosis rather than survival. This switch in roles for autophagy is thought to be dependent on the strength of the signals present, time of treatment and availability of ATP (57). At present our understanding of the type of death induced by autophagy, tends to reflect the greater understanding of apoptosis compared to necroptosis and autosis (58). Nevertheless, autophagy-induced apoptosis has been reported as an alternative fate of MSCs exposed to an inflammatory microenvironment (59). Dang et al. demonstrated that autophagy may cause cell death in a sepsis model of inflammation. These data suggest that the cytokine cocktail presented to the MSCs from the microenvironment causes autophagy to trigger death instead of promoting cell survival. This was mediated via the interaction with the ROS/ERK pathway resulting in the downregulation of Bcl-2. Inhibition of autophagy in MSCs led to increased production of prostaglandin E2 (55) and enhanced immunoregulation in pre-clinical models of EAE (55) and sepsis (59). Dang et al. (55) also reported that the induction of apoptosis reduced the therapeutic effect of MSCs, however, it has recently been demonstrated in a GvHD model that apoptotic MSCs are still immunosuppressive (7). Galleu et al. recorded that apoptotic MSCs (apoMSC) could reduce effector cell number in the lung and spleen of GvHD mice (7). Importantly, phagocytes producing indolamine 2,3-dioxygenase were required for the protection associated with apoMSC when administered intraperitoneally but not intravenously (60). These and other studies from the Hoogduijn group (5, 6) suggest that the therapeutic effects of both live and apoMSC are dependent on interactions with specific phagocytic cell populations. These observations also highlight the important interaction between MSCs and macrophages and the contribution of innate immune modulation to therapeutic efficacy (61). Given the contrasting data surrounding the effects on efficacy of autophagy in MSCs it is clear that further study is needed, especially of dose and temporal responses. Nevertheless, it is possible to state that the inflammatory environment plays an important role in the MSC fate of survival or death, that autophagic processes are involved in this fate decision, and that subsequent interaction of MSCs with innate cells such as monocytes/macrophages influence therapeutic efficacy. From the above studies, it seems likely that whereas pro-survival processes are likely to be linked in part to therapeutic effects through MSCs' production of paracrine factors (e.g., VEGF, etc.), necrobiological-related efficacy is more likely to operate through the interaction between MSCs and the innate immune cells such as monocytes/macrophages (6).

## Mitochondrial Transfer by MSCs and Clinical Efficacy

Cell death, oxidative stress, and autophagy are all linked to mitochondrial function (62), so it understandable that the mitochondrion has a role in MSC efficacy. More surprising have been the now well-documented reports that reprogramming of host cells by MSCs is significantly mediated by their ability to transfer functionally active mitochondria to somatic recipient cells. Mitochondrial transfer has been found to play a critical role in therapeutic effect of MSCs in the pre-clinical models of multiple diseases including brain injury, cardiac myopathies, muscle sepsis, and acute (ARDS) (63, 64) and chronic respiratory disorders (asthma and COPD) (65, 66). Mitochondria are transferred between cells via tunneling nanotubules (TNTs), cell fusion, and can also be contained in secreted extracellular vesicles (EV) (67). These mitochondria are functionally active and their transfer results in the enhancement of oxidative phosphorylation coupled with alleviation of oxidative stress in recipient cells leading to restoration of impaired functional activity (e.g., surfactant secretion, phagocytosis and wound healing) and cytoprotective effects. The consequences and mechanisms of mitochondrial transfer have been comprehensively reviewed previously (67–69). As mitochondrial dysfunction contributes to pathophysiology of various diseases, strategies aiming to protect mitochondria from injury or to increase biogenesis are being increasingly explored as promising therapeutic opportunities. Replacement of damaged mitochondria through donation from MSCs is a faster and physiologically more economical route for the recipient cell at the site of injury as compared to the mitochondrial biogenesis and therefore, appears to be an efficient means for disease attenuation (70).

In addition to protective effects due to improved bioenergetics, there is evidence of the involvement of mitochondrial transfer in cellular rejuvenation and transcriptional reprogramming (71). Studies by Acquistapace et al. demonstrated a key beneficial role of MSC mitochondria for reprogramming of post-mitotic murine cardiomyocytes toward proliferating cardiac progenitor-like cells through spontaneous cell fusion (72).

Although the precise mechanisms regulating mitochondrial extrusion from MSCs as well as their uptake by recipient cells remain to be investigated, it is clear that the injury microenvironment will have an impact on the rate and efficiency of this process. Thus, we have recently demonstrated that hypercapnia, a condition often associated with low tidal volume ventilation in ARDS, induces mitochondrial dysfunction and although the rate of mitochondrial transfer from MSCs to recipient cells is not changed, these dysfunctional mitochondria are not able to improve recipient cell bioenergetics and promote capacity of the lung epithelial cells to wound closure. This is in good agreement with the finding of Paliwal et al. demonstrating that mitochondria from MSCs with higher mitochondrial respiration capacities are more effective in suppression of mtROS in stressed recipient cells (73). Li et al. have demonstrated that pre-treatment with anti-oxidants such as N-acetyl-L-cysteine and L-ascorbic acid 2-phosphate enhanced mitochondrial transfer from the anti-oxidant treated population of the bone marrow derived MSCs to the untreated population of MSCs injured by H_2_O_2_ (74).

A key study by Mahrouf-Yorgov et al. reports that mitochondria released from dying cells at the site of injury are an important environmental cue that controls the cytoprotective function of MSCs and regulates their capacity for mitochondrial transfer (75). It was shown that upon oxidative stress, somatic cells (cardiomyocytes and endothelial cells) release mitochondria which are engulfed and degraded by MSCs, leading to induction of heme oxygenase-1 (HO-1) and stimulation of autophagy and mitochondrial biogenesis. As a result, the ability of MSCs to donate their mitochondria to injured cells to alleviate oxidative stress injury was enhanced (75). Reactive oxygen species and inflammatory cytokines (e.g., TNF-α) have also been postulated to play a role in the regulation of mitochondrial donation (67–69, 76). Our unpublished data suggest that mitochondrial transfer from MSCs to lung epithelial cells is enhanced in inflammatory environments. Taken together these data strongly suggest that mitochondrial transfer and cell death are related and relevant to clinical efficacy.

The processes of MSC mitochondrial transfer and autophagy are intrinsically interdependent. Phinney et al. have demonstrated that MSCs extrude their mitochondria in EVs which express autophagosomal markers, suggesting that this phenomenon is a result of incomplete autophagy (47). Physiologically, MSCs reside in the low oxygen environment of the bone marrow stem cell niche and the authors observed that conventional culture of MSCs in normoxia- (21% oxygen) induced oxidative stress, thereby promoting MSC mitophagy, however instead of degradation, mitochondria were directed outside of the cells (47). Ghanta et al. then demonstrated the importance of autophagy in maintaining healthy mitochondrial function and promoting survival in MSCs during oxidative stress (45).

In the view of accumulating evidence that after *in vivo* administration, MSCs undergo apoptosis and fragmentation and subsequent elimination by phagocytes (6, 7, 77), it is plausible to hypothesize that mitochondria could be released during fragmentation and taken up by surrounding somatic cells and particularly macrophages. We have previously demonstrated that mitochondrial transfer from healthy MSCs through extracellular vesicles results in macrophage metabolic reprogramming toward M2-like phenotype with enhanced phagocytic activity (63, 64) however whether or not mitochondria released from dying MSCs retain the same properties and exert similar effects remains to be determined.

## MSC-derived Extracellular Vesicles, and Clinical Efficacy

If there is a cell-specific therapeutic benefit from using MSCs as opposed to any apoptotic cell, then cell-specific characteristics and mechanisms need further exploration. Apoptotic cells maintain some biological activities as they begin the orderly process of disassembly and death. In particular apoptotic cells can produce a range of EVs and apoptotic bodies that can influence their microenvironment (78, 79). There has been an explosion in the literature describing how exosomes and other EVs can act as biological modulators. Healthy, viable MSCs are well-characterized producers of a wide range of EVs with different cargos. These can include microvesicles, including those bearing mitochondria (see above), as well as exosomes (66) that are now recognized as powerful mediators of intercellular communication locally and systemically. Exosomes, and presumably their cargo, can activate or suppress aspects of immunity such as cytokine secretion, immune cell differentiation and polarization, and T cell activation (47, 80–82). In addition, processes such as angiogenesis, proliferation, oncogenesis, and microenvironmental conditioning can all be affected by exosomes. MSC derived exosomes (even in the absence of their viable MSC producer) can thus have detectable therapeutic influences in human systems (Table 2). The influences of exosomes are largely defined by their cargo, which can include cytosolic and membrane proteins, mRNA and non-coding RNA including miRNA (miR), and the nature of the EV cargo of MSCs is influenced by the extracellular environment (Table 3). Several studies have linked treatment with MSC-derived exosomes to improvement in models of liver, kidney, heart, skin, lung and other diseases (90–93). The influence of MSC-derived exosomes on lung injury is especially important to studies of clinical efficacy given that the lung is a major site of MSC accumulation in the early period after delivery (94, 95). MSC-derived exosomes regulate vascular remodeling and reduce hypoxic pulmonary hypertension in rodent models. These exosomes reduced the activation of the hypoxic transcription factor STAT3 and the expression of the miRNA-17 superfamily but restored miRNA-204 in lung (normally reduced in human pulmonary hypertension) (83). In an acute respiratory distress syndrome (ARDS) model, alveolar macrophages treated with MSC-derived CD44^+^ EVs also reduced lung injury (64). As discussed above, EVs promoted mitochondrial transfer to the macrophages increasing their phagocytic capacity and inducing an anti-inflammatory response. These findings suggest that intravenously delivered MSC therapies that see an accumulation of viable pre-apoptotic MSCs in the lung vasculature, have the potential to produce EV with extensive biological effects.

**Table 2:** Selected studies of MSC-derived exosomes in human models.

**Experimental System**	**MSC**	**Cargo**	**Method of exosome isolation**	**Effect**	**Study**
PBMC co-culture	Bone marrow (healthy donors)	ND	Ultracentrifugation and precipitation	Suppressed TNF-a & IL-1b but increased anti-inflammatory factor TGF-b *in vitro*	(80)
PBMC co-culture	Bone marrow (healthy donors)	ND	Ultracentrifugation	Increased Treg/Teff ratio and IL-10 concentration in culture medium	(81)
Monocyte-derived macrophages	Bone marrow (healthy donors)	ND	Ultracentrifugation	Suppressed pro-inflammatory cytokine production, increased M2 macrophage marker expression, and augmented phagocytic capacity of human monocyte derived macrophages in non-contact cultures	(64)
Isolated human pulmonary artery endothelial cells	Umbilical cord	ND	S200 size-exclusion chromatography, differential centrifugation and ultracentrifugation	Regulated STAT3-mediated signaling	(83)
Human umbilical cord vein endo- thelial cells (HUVECs)	Bone marrow (healthy donors)	1,927 proteins identified	Differential centrifugation, filtration and ultracentrifugation	Proteomic analysis of proteins contained in exosomes released by MSC under ischemic like conditions. Mostly proteins such as platelet, epidermal or fibroblast derived growth factors, as well as proteins from nuclear factor-kappaB (NFkB) signaling pathway	(84)
HUVEC & human breast carcinoma-derived cell lines	Bone marrow (healthy donors)	miRNA-100	Differential centrifugation, filtration and ultracentrifugation	Decreased expression of VEGF in breast cancer-derived cells by modulating the mTOR/HIF-1α signaling axis	(85)
Comparative study	Bone marrow (healthy donors)	730 proteins identified in microvesicles	Sucrose cushion centrifugation & ultracentrifugation	Proteomic analysis identified proteins involved in cell proliferation, adhesion, migration, and morphogenesis	(86)

**Table 3:** Studies showing the influence of inflammatory environment on human MSC-derived exosome cargo.

**Stimulation**	**MSC**	**Cargo**	**Isolation/Treatment**	**Effect compared to control**	**References**
TNF-α + IFN-γ overnight	Human bone marrow derived MSC	ICAM 1, CXCL12, and CCL5. 11 miRNAs with direct or indirect immunomodulatory function	Tangential flow filtration	Stimulated MSC EVs increased anti-inflammatory response through COX2/PGE2 pathway modulation	(87)
TNF-α + IFN-γ overnight	Human bone marrow derived MSC	ND	Tangential flow filtration	Improved mechanical sensitivity in rat spinal cord injury model	(88)
TGF-β, IFN-γ, or TGF-β + IFN-γ for 72 h	Human umbilical cord derived MSC	Exosomes from MSC treated with TGF-β and IFN-γ contained more IFN-γ, IL-10, and IDO	Centrifugation and PEG6000	EV from MSCs treated with TGF-β and IFN-γ induced Tregs differentiation	(89)

Perhaps the most profound influence of MSC-derived exosomes and EVs is the regulation of innate immune responses. Phinney et al. showed that MSC-derived exosomes with miRNA cargo inhibited macrophage activation by modulating Toll-like receptor signaling (47, 96). Macrophages treated with MSC-derived exosomes activated NF-kB and changed the expression of 50 of the 84 TLR-associated proteins evaluated, including IL-1β, COX2, IL-10, CCL2, TNF, MyD88, TLR 1,4,5,7,8 and 9, IRAK1, and TRAF6 (47). The breadth of biological processes downstream of these factors is very extensive and hints at the potential scale of effects that might be influenced by exosomes produced in the early, pre-apoptotic, phase of MSC therapy. In adaptive immunity (and hence of relevance to cell therapy for autoimmune disease and transplantation), EVs from bone marrow-derived MSCs increased production of immunosuppressive IL-10 and the proliferation of regulatory T cells in peripheral blood mononuclear cell cultures stimulated with anti-CD3/CD28 beads (81). In this research, treatment with MSC-EVs alone resulted in apoptosis of T cell populations. Interestingly, in other studies, exosomes secreted by HIF-1α-overexpressing donor MSCs were enriched for the Notch ligand Jagged-1 (97). Subcutaneous injection of these exosomes in a Matrigel plug assay induced angiogenesis (97). This builds on earlier work showing that Jagged-1 was an important contact dependent signal by which MSCs induced tolerogenic dendritic cells (DC) (98). Given that DCs have an antigen acquisition sentinel function throughout the body, and that these are key cells in shaping adaptive immunity, exosomal cargos of Notch ligands might prove an important modulator of immunity in multiple cell therapies. The immunomodulatory properties of MSC-derived subcellular particles indicate their potential as a novel cell-free therapy for treatment of immunological disorders, especially through interaction with antigen presenting cells (61). This is borne out by a recent study showing differential effects of membrane derived particles from MSCs either untreated or pre-treated with IFN-γ. Whilst both particle types decreased the frequency of CD14^+^ CD16^+^ inflammatory monocytes, the particles derived from IFN-γ treated cells also promoted anti-inflammatory PD-L1 expressing monocytes (10). This provides a mechanistic basis for earlier work showing that IFN-γ does not break but enhances the immunosuppressive capacities of MSCs and MSC-like cells (26, 99, 100). In the context of necrobiology, these data indicate that pre-apoptotic MSCs used as therapies in inflammatory microenvironments could be responsible for a switch toward an anti-inflammatory response through subcellular particles through their intra-vesicular or surface cargo (10).

The second aspect of MSC necrobiology that could affect therapeutic efficacy is the very recent observation from tumor biology that apoptotic cancer cells produce EVs with the characteristics of exosomes (78, 101). Apoptotic cell-derived extracellular vesicles (apoEVs) appear to be enriched with snRNA and spliceosomal proteins that can alter mRNA splicing in recipient cells (101). This finding is consistent with other studies showing that EVs produced during apoptosis are not simply debris but have important immune regulatory roles in autoimmunity, infection and cancer (78, 79). Thus, apoEVs including those with exosome characteristics are the conduit of intercellular communication in physiologic and pathologic contexts. In this regard, it is important to note that there has yet to be a comprehensive description of exosomes produced by apoptotic MSCs. We do not know the degree to which apoptotic MSCs produce apoEVs, nor how this is affected by MSC history, stimulation, source, or apoptotic stage. Nevertheless, it is clear that MSC-derived subcellular particles' contents are not static but vary by tissue origin, MSC activity, and the cellular environment of the MSCs (96). It remains reasonable to assume that apoptotic MSCs produce apoEVs with potential to modify target cells.

Overall, the important implication is that MSCs (whether viable or non-viable) delivered to a patient are likely to be accompanied by or result in EV with diverse cargo produced prior to or after therapeutic deployment. However, it is also worth remembering that broader animal studies of MSCs that do not consider exosome function, could be unwittingly measuring a confounding effect of bovine exosomes present in the serum constituents of culture medium. This is usually well-controlled for in studies designed to discover exosome effects, but less often in studies of the MSC function itself. The range of such effects are extensive and could be influencing multiple disease models (Table 2). Nonetheless, the beneficial effects of exosomes derived from various sources has led to over 100 human phase I/II clinical trials, although to date there are very few reports of trials involving human MSC derived exosomes (www.clinicaltrials.gov). Those that have been registered target pancreatic cancer, macular holes, cerebrovascular disorders and diabetes, but most seem to be in the recruitment phase at present.

## Conclusion

A common aspect of all the above aspects of MSC necrobiology is the significant role played by innate immune cells to counter the pathologic processes. Thus, efferocytosis or the processes linked to removing apoptotic MSCs are likely to contribute to the therapeutic benefit in studies where MSC viability is not essential (2, 102). This is likely to extend beyond simple uptake of apoptotic MSCs by macrophages, dendritic or other cells (61), and extend to the range of EVs, mitochondria, and other signals produced by dying MSCs and which profoundly alter the tissue microenvironment and innate immune cells (8, 102). The importance of these processes in the regular homeostatic function of endogenous MSCs is not known. Nevertheless, in the context of cell therapy, the efficacy of MSC can be attributed to either live/viable or dying/dead MSCs in different disease contexts, and these benefits are attributable to downstream effects linked to: a) the biological activity to (or evoked by) the intended therapeutic component (the viable MSC itself or its derivatives) and/or b) the recipient's response to MSCs that are in the process of dying (Figure 1). Without this understanding, and a greater appreciation of the complex necrobiology of MSCs, we are unlikely to understand the mechanisms of cell therapy action or rationally design improvements. Thus, the necrobiology of the mesenchymal stromal cell is likely to be a fruitful area for improving the efficacy or removing confounding influences on cell therapy.

## Author Contributions

DW has performed literature research, designed the review layout, wrote, and revised the review. KE has performed literature research, designed the review layout, wrote, and revised the review. AK has performed literature research, designed the review layout, wrote, and revised the review. JI-C has performed the literature research and contributed to the section on extracellular vesicles. IH has performed the literature research and contributed to the section on autophagy. BM has performed literature research, designed the review layout, wrote, and revised the review. All authors agree to be accountable for the content of the work.

### Conflict of Interest Statement

The authors declare that the research was conducted in the absence of any commercial or financial relationships that could be construed as a potential conflict of interest.
